# Photocurrent response and semiconductor characteristics of Ce-Ce_2_O_3_-CeO_2_-modified TiO_2_ nanotube arrays

**DOI:** 10.1186/1556-276X-9-67

**Published:** 2014-02-10

**Authors:** Yu Tan, Shenghan Zhang, Kexin Liang

**Affiliations:** 1 School of Environment Science and Engineering, North China Electric Power University, Yonghua North Street 619#, Baoding 071003, China

**Keywords:** TiO_2_ nanotube arrays (TNTs), Ce, Photocurrent response, Semiconductor characteristic, Nanocomposites

## Abstract

We reported Ce and its oxide-modified TiO_2_ nanotube arrays (TNTs) and their semiconductor properties. The TNTs were prepared by anodic oxidation on pure Ti and investigated by electrochemical photocurrent response analysis. Then, the TNT electrodes were deposited of Ce by cathodic reduction of Ce(NO_3_)_3_ 6H_2_O. After deposition, the TNT electrodes were fabricated by anodic oxidation at *E* = 1.0 V_(SCE)_ for various electricity as Ce-Ce_2_O_3_-CeO_2_ modification. The Ce-deposited TNTs (band gap energy *E*_g_ = 2.92 eV) exhibited enhanced photocurrent responses under visible light region and indicated more negative flat band potential (*E*_fb_) compared with the TNTs without deposition. After anodic oxidation, the mixed Ce and its oxide (Ce_2_O_3_-CeO_2_)-modified TNT photoelectrodes exhibited higher photocurrent responses under both visible and UV light regions than the TNTs without deposition. The photocurrent responses and *E*_fb_ were found to be strongly dependent on the contents of Ce_2_O_3_ and CeO_2_ deposited on TNTs. A new characteristic of *E*_g_ = 2.1 ± 0.1 eV was investigated in the Ce_2_O_3_- and CeO_2_-modified photoelectrodes. X-ray diffraction (XRD), scanning electron microscopy (SEM), and X-ray photoelectron spectroscopy (XPS) were also employed to characterize various modified TNTs photoelectrodes.

## Background

One-dimensional TiO_2_ nanotubes arrays (TNTs) can provide higher surface area [1] and higher interfacial electricity transfer rate rather than spherical particles [2]. TNTs have been modified by deposition of metal or metal oxides [3,4] to indicate an enhanced photoelectric response under visible light. Nowadays, the rare earth metal Ce with *f* electron distribution has received extensive attention [5] for its energy levels located in the forbidden band of TiO_2_ which can form additional levels to accelerate the separation of electrons and holes [6]. The different electronic structures of Ce^3+^ with 4*f*^1^5*d*^0^ and Ce^4+^ with 4*f*^0^5*d*^0^ indicate different optical properties [7-10]. The oxides of Ce indicate different semiconductor characteristics such as Ce_2_O_3_, with narrow bandgap energy (*E*_g_ = 2.4 eV), which is able to absorb visible light and CeO_2_, with wide bandgap energy (*E*_g_ = 3.16 eV), which can strongly absorb UV light even better than TiO_2_[11]. The redox couple of Ce^3+^/Ce^4+^ can shift between CeO_2_ and Ce_2_O_3_ during oxidizing and reducing process [12]. Li et al. [13] reported higher adsorption equilibrium constant and higher separation efficiency of electron-hole pairs obtained simultaneously from Ce^3+^-TiO_2_ catalysts. Due to the less acknowledgement of behavior of Ce and its oxides, the researches about Ce and its oxide deposition on TNTs are uncommon.

In this study, different proportions of Ce mixtures (Ce, CeO_2_, and Ce_2_O_3_) deposited TNTs were prepared to investigate their photocurrent responses and semiconductor characteristics.

## Methods

Prior to anodization, the titanium sheets were mechanically polished with different abrasive papers and ultrasonically degreased in acetone and ethanol, respectively, finally rinsed with deionized water and dried in air. All the anodization experiments were carried out in a conventional two-electrode electrochemical cell under magnetic agitation condition at room temperature, with titanium foil as the anode and platinum foil as the cathode. The ethylene glycol solution containing 0.5 wt.% NH_4_F and 1.5 vol% H_2_O was used as electrolyte. The anodization voltage was constant at 20 V with a direct current power supply. The anodization process was performed for 6 h to obtain TNTs. After electrochemical anodization, the as-anodized TNTs were immediately rinsed with deionized water and then dried at 100°C. All samples were annealed at 450°C for 1.5 h to transform amorphous TiO_2_ to crystalline phase.

Firstly, the reductive Ce-deposited TNTs were performed by electrochemical reduction. The as-prepared TNTs with exposed area 0.2826 cm^2^ were inserted in 0.01 M Ce(NO)_3_ · 6H_2_O alcohol electrolyte for 1 h adsorption. Then, the above TNTs were used as working electrode, a Pt foil as the anode, and a saturated calomel electrode (SCE) as the reference electrode in the electrolyte. A potential *E* = -6 V was applied in the three-electrode system until a total electricity *Q* = 0.01 C to reduce Ce^3+^ into elemental Ce deposition on TNTs. This modified sample was named as TNTs-Ce. Secondly, several TNTs-Ce samples were oxidized by potentiostat powered by an anodic potential *E* = 1.0 V to the sample in supporting electrolyte (0.01 M Ce(NO_3_)_3_) for total electricity *Q* = 0.00001, 0.00025, 0.005, and 0.01 C, respectively. The oxidized samples were denoted as TNTs-0.00001 C, TNTs-0.00025 C, TNTs-0.005 C, and TNTs-0.01 C, correspondingly.

The morphologies were observed using field emission scanning electron microscope (FE-SEM, JSM-7500 F) with energy dispersive X-ray spectroscopy (EDX). The crystal phases and composition were characterized by X-ray diffraction (XRD, Y-2000) and X-ray photoelectron spectroscopy (XPS, MT-500, with Al monochromator with C1s at 284.8 eV). The photocurrent response measurements were carried out in an improved three-electrode electrochemical cell with a quartz window and 0.1 M Na_2_SO_4_ as supporting electrolyte. A 450-W Xeon lamp, a CT110 monochromator (1/8, Crowntech), and a potentiostat (PARSTAT2273, Princeton Applied Research, Oak Ridge, TN, USA) were also applied for electrochemistry measurements. The Mott-Schottky plots were performed with frequency 1,000 Hz and applied potential from -1.0 to 0.5 V by 0.1 V steps.

## Results and discussion

**Figure 1:**
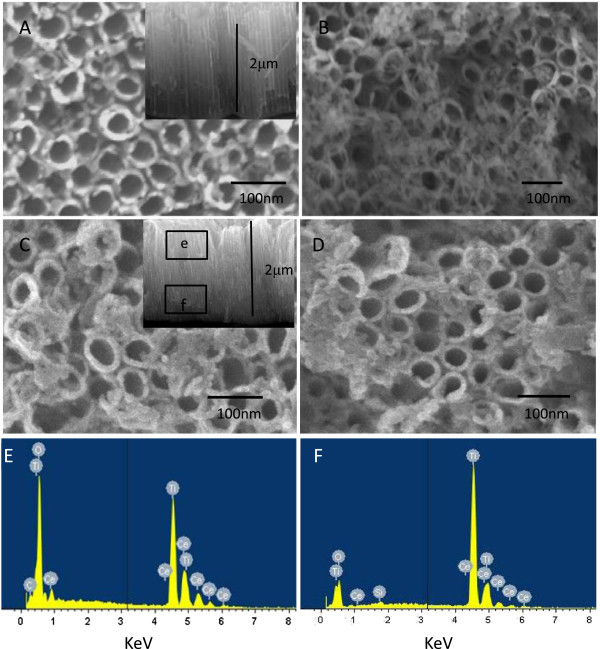
**SEM images.** Of **(A)** TNTs with inset cross section image, **(B)** TNTs-Ce, **(C)** TNTs-0.00025 C with inset cross section image, **(D)** TNTs-0.01 C, **(E)** and **(F)** corresponding EDX spectra of e and f in **(C)**.

Figure 1 shows the SEM images of the (A) TNTs, (B) TNTs-Ce, (C) TNTs-0.00025 C, and (D) TNTs-0.01 C. Figure 1A indicates an average diameter of 50 nm and tube length of 2 μm of TNTs. After deposition, the morphology of the TNTs was changed by reductive Ce or oxidative Ce. Cross section SEM and EDX are also employed to confirm the decoration of Ce in the tubes from Figure 1C,D,E,F. From the EDX spectra, the nanotubes near the top contained more Ce (Ti/Ce = 3.17) than the nanotubes near the bottom (Ti/Ce = 10.98).

**Figure 2:**
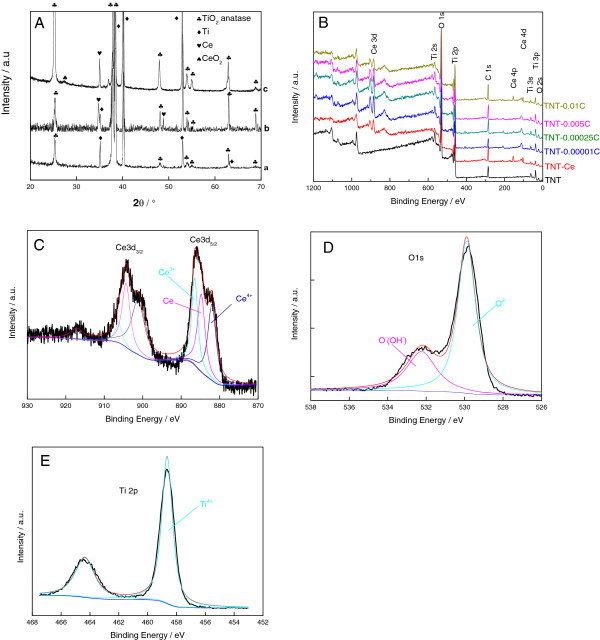
**XRD patterns and XPS spectrum survey. (A)** XRD patterns for (a) TNTs, (b) TNTs-Ce, and (c) TNTs-0.00025 C. **(B)** XPS spectrum survey of various samples. XPS spectrum of **(C)** Ce3d, **(D)** O1s, and **(E)** Ti2p of TNTs-0.00025 C.

According to XRD patterns in Figure 2A, TNTs indicate anatase crystal phase. The simple substance Ce can be identified on TNTs-Ce. After anodic oxidation, the elemental Ce and CeO_2_ are detected in the deposited materials. They agree well with the reported values from JPCDS card (TiO_2_ 73-1764), (Ti 44-1294), (Ce 38-0765), and (CeO_2_ 44-1001).

Figure 2B shows the survey of various samples, and Figure 2C,D,E shows the XPS spectra of TNTs-0.00025 C. The characteristic peaks of Ce3d are splitted to multipeak structure and fitted according to reference [14], besides O1s and Ti2p. The oxidative Ce is a mixture of Ce, Ce_2_O_3_, and CeO_2_. The relative proportions are calculated from the fitting data as Table 1. According to the table, quantification of simple substance Ce decreases as the oxidation of electricity increases. During the oxidation process, the Ce_2_O_3_ and CeO_2_ increases as the electricity increases. It should be highlighted that the existence of Ce_2_O_3_ and CeO_2_ in TNTs-Ce which indicated that the reduction process contribute not only the reduced state of Ce but also the oxidation state. Apparently, the ration of Ti/Ce increases as the oxidation of electricity increases. The tendency of Ti/O is not clear.

**Table 1:** Ratio of Ce in various photoelectrodes calculated from XPS analysis

	**Ce**	**Ce**_**2**_**O**_**3**_	**CeO**_**2**_	**Ti/Ce**	**Ti/O**
TNTs					0.43
TNTs-Ce	71.6	6.70	21.6	3.57	0.19
TNTs-0.00001 C	57.3	13.3	29.4	3.78	0.30
TNTs-0.00025 C	33.7	33.6	32.6	3.89	0.28
TNTs-0.005 C	28.4	36.7	34.9	5.34	0.31
TNTs-0.01 C	16.1	42.0	41.9	5.56	0.23

Values in at.%.

**Figure 3:**
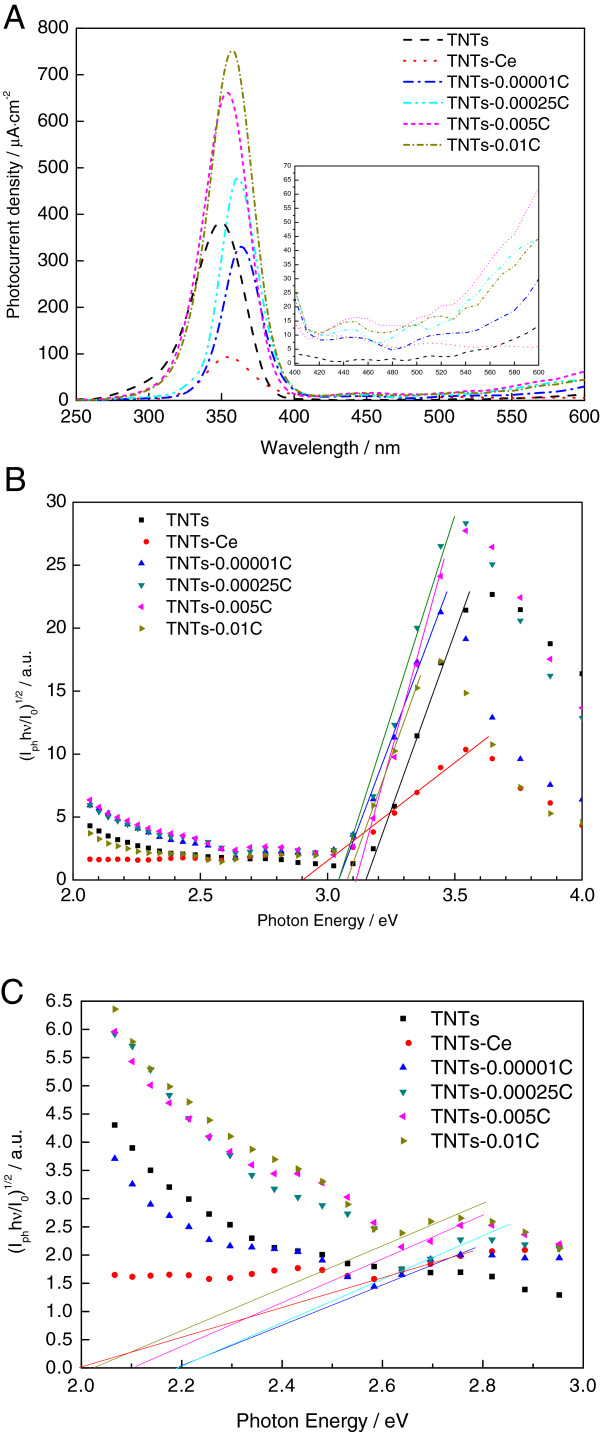
**Photocurrent analysis results. (A)** Photocurrent responses vs. wavelength plots. **(B)** Photocurrent responses vs. photon energy plots. **(C)** Low photon energy part of Figure 3B (from 2.0 to 3.0 eV).

The photocurrent spectra vs. wavelength are showed in Figure 3A. The TNTs-Ce indicates stronger photocurrent response in visible light region and weaker photocurrent response in UV light region compared to the TNTs without deposition. After anode oxidation, Ce-Ce_2_O_3_-CeO_2_ modification photoelectrodes showed stronger photocurrent response in visible. In UV light region, the photocurrents responses of the photoelectrodes are reinforced as oxidation electricity increases with CeO_2_ increasing except TNTs-0.00001 C. The reason could be as followed: the Ce^4+^ is an efficient electron acceptor during the photocurrent production. But the deposition of Ce and its oxide affect the surface morphology of TNTs (Figure 2B) which reduced the absorption of light. In visible light region as the oxidation in depth with Ce_2_O_3_ is increasing, firstly, the photocurrent responses of the TNTs-0.00001 C, TNTs-0.00025 C, and TNTs-0.005 C are gradually increased; then, the photocurrent response of TNTs-0.01 C is slightly decreased by Ce_2_O_3_ transfer to CeO_2_.

The relationship between photocurrent *I*_ph_ and bandgap energy *E*_g_ of the oxide films on alloys can be written in the form [15]:

(1) $${\left(I_{\mathrm{ph}}h\nu /I_{0}\right)}^{1/n}=A\;\left(h\nu -E_{g}\right)$$

where *I*_0_, hv, *E*_g_, *A*, and *n* are fully discussed in [15] and *n* = 2 for the indirect transition of semiconductors. Figure 3B shows the photocurrent responses vs. photon energy plots for TNTs with various Ce deposits. Based on linear fitting, the characteristic *E*_g_ of various photoelectrodes can be derived respectively. *E*_g_ of the TNTs-Ce is reduced to 2.92 eV. After anodic oxidation, all the samples are located in the *E*_g_ between 3.0 to 3.1 eV, which are smaller than *E*_g_ of TNTs (3.15 eV) as a result of simple substance Ce existence.

Figure 3C shows the details of low electron energy part of Figure 3B. The various Ce-deposited TNTs indicated *E*_g_ of 2.1 ± 0.1 eV which is close to the *E*_g_ = 2.4 eV of Ce_2_O_3_. And these differences may be caused by the deposition of the simple substance Ce.

**Figure 4:**
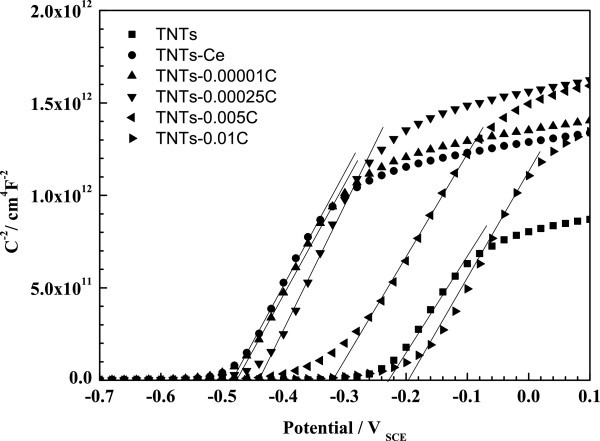
**Mott-Schottky plots of all the samples in 0.1 M Na**_**2**_**SO**_**4**_**, with frequency 1,000 Hz.**

Figure 4 shows the Mott-Schottky plots for various TNT photoelectrodes. The intercept of the straight line of Mott-Schottky plot at the potential axis corresponds to *E*_fb_ as listed in Table 2. The *E*_fb_ of TNTs-Ce moves to negative potential compared to TNTs, which infers the reducibility of electrons in TNTs-Ce excited to conduction band enhanced [16]. With the oxidation of Ce in depth, the *E*_fb_ moves to positive potential. But all the Ce oxide-modified TNTs' *E*_fb_ are negative to TNTs except the TNTs-0.01 C.

**Table 2:** Flat band potentials calculated from Mott-Schottky plots

	**TNTs**	**TNTs-Ce**	**TNTs-0.00001 C**	**TNTs-0.00025 C**	**TNTs-0.005 C**	**TNTs-0.01 C**
*E*_fb_/*V*	-0.24	-0.49	-0.48	-0.45	-0.33	-0.20

## Conclusions

Ce-modified TNTs indicated stronger photocurrent response in visible light and less noble flat band potential than TNTs. After anodic oxidation, the Ce-Ce_2_O_3_-CeO_2_-modified TiO_2_ nanotube arrays indicated higher photocurrent responses in both visible and UV light region. As the anodic oxidation in depth with Ce_2_O_3_ and CeO_2_ was increasing, the photocurrent responses reinforced, but the flat band potential moved to noble potential comparing to the TNTs-Ce. A characteristic *E*_g_ = 2.1 ± 0.1 eV in line with Ce_2_O_3_ was discovered from the photocurrent responses which increased the photocurrent responses in visible light region.

## Competing interests

The authors declare that they have no competing interests.

## Authors’ contributions

YT carried out the TiO_2_ nanotube arrays preparation, photoelectrochemical investigation, and SEM/XPS analysis. SZ carried out the Mott-Schottky plots analysis and calculation. KL wrote and designed the study. All authors read and approved the final manuscript.
